# Keratite infectieuse sous lentilles de contact

**DOI:** 10.11604/pamj.2014.17.276.4123

**Published:** 2014-04-14

**Authors:** Belmokhtar Adil, Daoudi Rajaa

**Affiliations:** 1 Université Mohamed V souissi, hôpital des Spécialités, Département d’ Ophtalmologie A

**Keywords:** Keratite, infection, lentilles de contact, keratitis, infection, contact lens

## Image en medicine

Les kératites infectieuses chez les porteurs de lentilles de contact sont de plus en plus fréquentes et constituent une véritable urgence diagnostique et thérapeutique. Les facteurs de risques sont essentiellement: la mauvaise hygiène, le port permanent, la contamination de solutions d'entretien. Nous rapportons un cas d'une patiente âgée de 40 ans qui a consulté pour une baisse d'acuité visuelle unilatérale suite à un port de lentilles colorés cosmétique. L'examen objective une acuité visuelle à mouvement des doigts à l'oeil droit, une hyperhémie conjonctivale avec cercle périkératique. La cornée est siège d'un infiltrat stromal sous jacent à une ulcération épithéliale de 3mm centrale (A). La chambre antérieure siège d'un tyndall inflammatoire (2++). Le reste de l'examen était inaccessible. Notre conduite a été d'hospitaliser la patiente, de réaliser un grattage cornéen à l'aide du vaccinostyl pour l'examen direct ainsi que la culture afin d'adapter l'antibiothérapie. Une antibiothérapie à large spectre a été démarrée, à base de collyres fortifiés (Voncomycine et le Fortum). La culture a mis en évidence un pseudomonas et l'antibiothérapie a été adapté. L’évolution a été marquée par l'amendement des signes inflammatoires et infectieuses, néanmoins, la persistance d'une taie cornéenne centrale a grevé la fonction visuelle (B). L'abcès de cornée présente une complication dramatique chez les porteurs de lentilles de contact. La prévention doit passer par une information et une sensibilisation des patients présentant des risques potentiels d'infection ainsi qu'une réglementation de la vente des lentilles cosmétiques.

**Figure 1:**
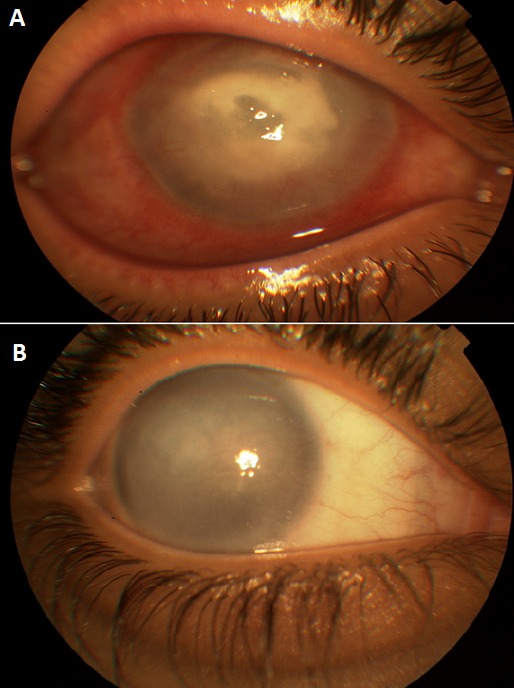
A) infiltrat stromal sous jacent à une ulcération épithéliale; B) Evolution après traitement

